# Extended Foville Syndrome Due to a Pontine Tegmental Hemorrhage: A Clinicoradiologic Case Report

**DOI:** 10.7759/cureus.115834

**Published:** 2026-09-05

**Authors:** Diana P Sy, Jon Stewart H Dy

**Affiliations:** 1 Neurology, Chinese General Hospital and Medical Center, Manila City, PHL; 2 Neurosciences, Mapua University School of Medicine, Makati City, PHL; 3 Neurosciences, St. Luke's Medical Center College of Medicine - William H. Quasha Memorial, Quezon City, PHL

**Keywords:** clinicoradiologic correlation, foville syndrome, intraparenchymal hemorrhage (iph), pontine hemorrhagic stroke, pontine tegmentum

## Abstract

Foville syndrome is a rare pontine tegmental syndrome classically characterized by ipsilateral abducens and peripheral facial nerve palsies with contralateral hemiparesis. Extended variants involving additional ocular motor, sensory, and autonomic pathways are uncommon, particularly when caused by hemorrhagic stroke. We report a 59-year-old Filipino male with uncontrolled hypertension who presented within hours of symptom onset with left lateral rectus paresis, left peripheral facial palsy, moderate dysarthria, right hemiparesis, and mild right hemisensory loss, with a National Institutes of Health Stroke Scale (NIHSS) score of 7. Non-contrast computed tomography demonstrated a focal left pontine tegmental hemorrhage. On the first hospital day, the left abducens deficit progressed to complete palsy, the right-sided sensory deficit worsened, and left internuclear ophthalmoplegia and Horner’s syndrome emerged, with an increase in NIHSS to 9. The patient was managed conservatively and underwent rehabilitation. At 1.5 months postictus, he was ambulatory with assistance with a modified Rankin Scale (mRS) score of 3. At 8 months, his NIHSS had improved to 6 and his mRS to 2, with independent ambulation despite residual focal neurological deficits. This case demonstrates the value of serial neurological examination and clinicoradiologic correlation in localizing evolving pontine tegmental lesions and illustrates meaningful functional recovery despite persistent deficits following focal pontine hemorrhage.

## Introduction

Foville syndrome is a rare crossed pontine syndrome resulting from lesions of the dorsal pontine tegmentum. The pons contains closely situated cranial nerve nuclei and fascicles, horizontal gaze pathways, and long motor and sensory tracts; consequently, a focal lesion may produce ipsilateral cranial nerve or gaze abnormalities together with contralateral limb deficits [1,2]. Classical Foville syndrome involves the abducens nucleus and/or adjacent paramedian pontine reticular formation (PPRF), producing an ipsilateral conjugate horizontal gaze palsy, often accompanied by ipsilateral peripheral facial nerve palsy and contralateral hemiparesis [1,2]. This nuclear or gaze-center involvement differs from an isolated abducens fascicular lesion, which produces impaired abduction of the ipsilateral eye rather than loss of conjugate horizontal gaze. Ipsilateral abducens nerve palsy with contralateral hemiparesis is more characteristic of Raymond syndrome, while additional involvement of the facial nerve may occur in Millard-Gubler syndrome. The close anatomical relationship of these structures explains why focal pontine lesions may produce overlapping crossed pontine syndromes and complex but highly localizing neurological deficits [1,2].

Extended variants of Foville syndrome may occur when adjacent pontine structures are involved. Extension to the medial longitudinal fasciculus may result in internuclear ophthalmoplegia, while disruption of descending hypothalamospinal sympathetic fibers may produce ipsilateral Horner’s syndrome [2]. Contralateral sensory deficits may accompany involvement of ascending sensory pathways [2]. These additional findings broaden the classical syndrome and may complicate differentiation from other crossed pontine syndromes.

Foville syndrome may result from vascular, demyelinating, neoplastic, inflammatory, or infectious lesions of the pons [2]. Among vascular etiologies, focal pontine hemorrhage represents an important cause because the clinical manifestations depend closely on the size and anatomical distribution of the hematoma [3]. Careful correlation between serial neurological findings and neuroimaging is therefore particularly valuable in establishing the anatomical extent of pontine involvement.

We report a case of extended Foville syndrome secondary to a focal pontine tegmental hemorrhage, characterized by evolving ocular motor, autonomic, motor, sensory, and speech deficits. The case demonstrates how serial neurological examination can refine brainstem localization as additional signs emerge and provides longitudinal clinical and functional outcomes through 8 months postictus.

## Case presentation

A 59-year-old Filipino male with a history of uncontrolled hypertension was brought to the emergency department within hours of acute symptom onset, presenting with binocular diplopia, left-sided facial weakness, and right-sided weakness and sensory loss. He had no prior history of cerebrovascular disease, diabetes mellitus, dyslipidemia, cardiac disease, or other relevant neurological conditions. There was no history of trauma. The patient denied smoking, alcohol intake, or illicit drug use.

On arrival, his blood pressure was 200/100 mmHg. Heart rate, respiratory rate, oxygen saturation, and temperature were within normal limits. Cardiovascular, respiratory, and abdominal examinations were unremarkable.

On initial neurological examination, the patient was awake, alert, and oriented. In primary gaze, the left eye was esotropic, while the right eye remained in the midline. Cranial nerve examination demonstrated left lateral rectus paresis, consistent with a left abducens nerve deficit. Vertical gaze was intact. A left lower motor neuron facial palsy was also present, characterized by weakness of the entire left hemiface. Pupillary size and reactivity were initially symmetric. Moderate dysarthria was noted. Motor examination demonstrated right-sided hemiparesis involving both the upper and lower extremities. Sensory examination revealed mild reduction of pinprick and light-touch sensation over the right side of the body. Deep tendon reflexes were brisk on the right, and the plantar response was extensor on the right. The National Institutes of Health Stroke Scale (NIHSS) score on admission was 7 [4].

Non-contrast computed tomography (CT) of the brain performed on admission demonstrated a focal intraparenchymal hemorrhage localized to the left pontine tegmentum, measuring approximately 3.0 cc. Minimal surrounding perilesional edema produced minimal compression of the fourth ventricle, without ventricular dilatation, intraventricular extension, or midline shift. Chronic small-vessel ischemic changes, mild cerebral atrophy, and atherosclerotic changes involving the internal carotid and vertebral arteries were also noted (Figure 1).

**Figure 1:**
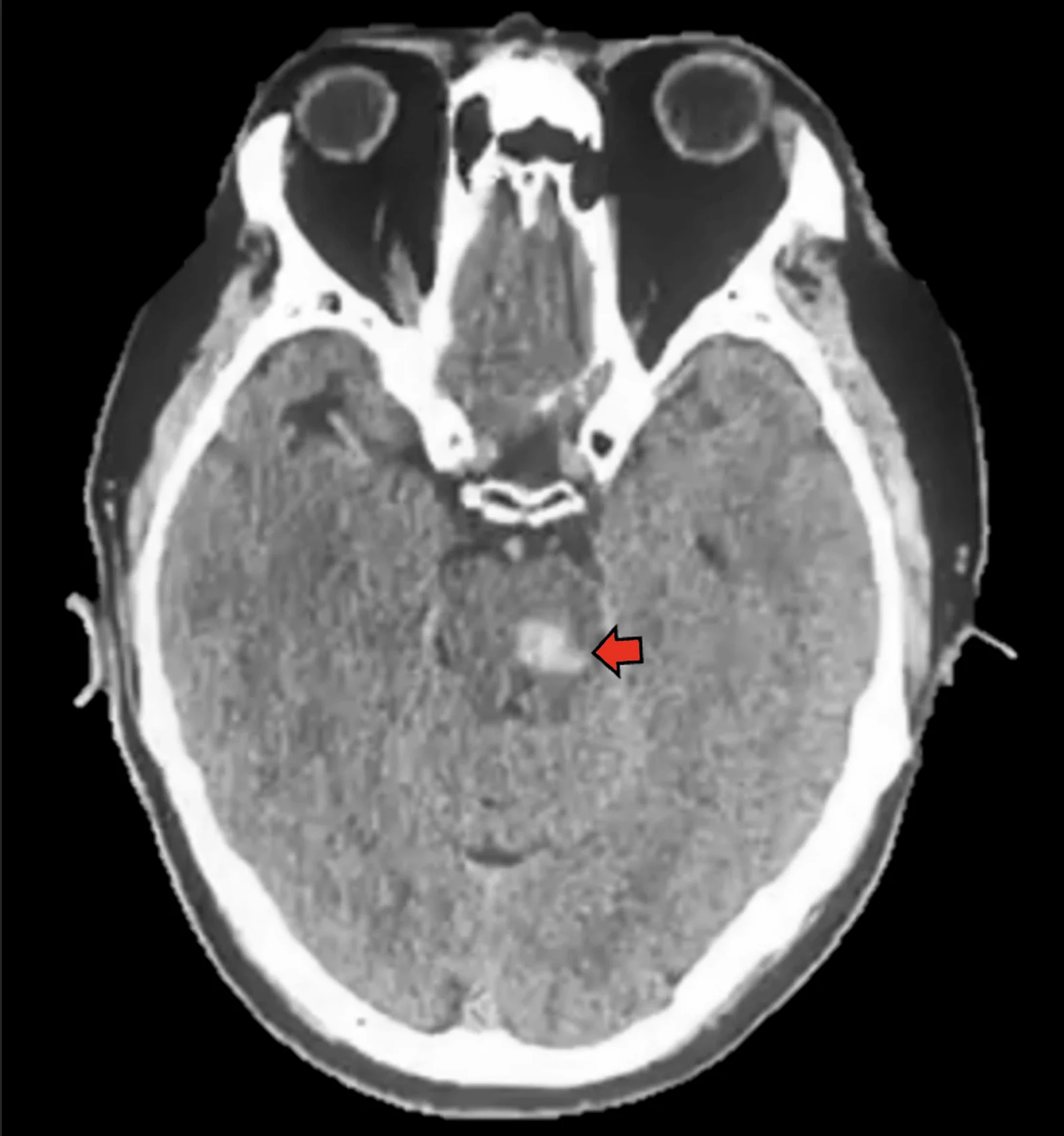
Non-contrast axial computed tomography of the brain demonstrates a focal hyperdense intraparenchymal hemorrhage (red arrow) within the left pontine tegmentum, without intraventricular extension or hydrocephalus.

Routine laboratory investigations, including complete blood count, serum electrolytes, renal function tests, liver enzymes, fasting blood glucose, glycohemoglobin, lipid profile, and coagulation studies, were obtained (Table 1). There was no laboratory evidence of coagulopathy or significant metabolic derangement that could account for the hemorrhage. Leukocytosis with neutrophilia was noted, with a white blood cell count of 17,400 cells/mm³ and neutrophil proportion of 84%; there was no clinical evidence of infection. Aspartate aminotransferase (AST) was elevated at 70 U/L, while alanine aminotransferase (ALT) was within the reference range at 33 U/L. Fasting blood glucose was 99.82 mg/dL, and glycohemoglobin was 5.7%. Lipid profile demonstrated hypertriglyceridemia at 246.90 mg/dL and elevated very-low-density lipoprotein cholesterol at 49.03 mg/dL, with total cholesterol of 103.47 mg/dL, high-density lipoprotein cholesterol of 42.08 mg/dL, and low-density lipoprotein cholesterol of 12.36 mg/dL.

**Table 1:** Laboratory investigations obtained during admission Leukocytosis with neutrophilia, elevated AST, hypertriglyceridemia, and elevated VLDL-C were noted. Prothrombin time and INR showed no evidence of coagulopathy. *Laboratory-provided LDL-C reference target for individuals aged ≥45 years without diabetes mellitus or atherosclerotic cardiovascular disease and with ≥2 risk factors. g/dL = grams per deciliter; mm3 = cubic millimeters; fL = femtoliter; mg/dL = milligrams per deciliter; mmol/L = millimoles per liter; U/L = units per liter

Test	Result	Reference Value
Hematology		
Hemoglobin	15.6 g/dL	11.6–15.5 g/dL
Hematocrit	47.60%	36.0–47.0%
White blood cell count	17,400/mm³	4,800–10,800/mm³
Neutrophils	84%	40–74%
Lymphocytes	10%	19–48%
Monocytes	5%	3–9%
Platelet count	281,000/mm³	150,000–400,000/mm³
Mean corpuscular volume	94.2 fL	82–98 fL
Mean corpuscular hemoglobin	30.8 pg	28–33 pg
Mean corpuscular hemoglobin concentration	32.70 g/dL	32–38 g/dL
Renal Function and Electrolytes		
Creatinine	0.75 mg/dL	0.55–1.20 mg/dL
Blood urea nitrogen	3.4 mg/dL	9–23 mg/dL
Sodium	136 mmol/L	136–145 mmol/L
Potassium	4.2 mmol/L	3.5–5.1 mmol/L
Liver Enzymes		
Alanine aminotransferase (ALT)	33 U/L	10–49 U/L
Aspartate aminotransferase (AST)	70 U/L	0–34 U/L
Glycemic Profile		
Fasting blood glucose	99.82 mg/dL	79–115 mg/dL
Glycohemoglobin (HbA1c)	5.70%	3.5–6.0%
Lipid Profile		
Total cholesterol	103.47 mg/dL	<240 mg/dL
Triglycerides	246.90 mg/dL	≤150 mg/dL
High-density lipoprotein cholesterol (HDL-C)	42.08 mg/dL	>40 mg/dL
Low-density lipoprotein cholesterol (LDL-C)	12.36 mg/dL	<130 mg/dL*
Very-low-density lipoprotein cholesterol (VLDL-C)	49.03 mg/dL	0–40 mg/dL
Total cholesterol/HDL-C ratio	2.46	<4.0
Coagulation Profile		
Prothrombin time (PT)	14.5 seconds	11.7–15.3 seconds
International normalized ratio (INR)	1.07	—
Prothrombin activity	90.00%	—

On the first hospital day, the patient demonstrated progression of his neurological deficits. The initial left lateral rectus paresis progressed to complete left lateral rectus palsy, with loss of abduction of the left eye, while sensory loss over the right upper and lower extremities became more pronounced. He also developed left internuclear ophthalmoplegia, manifested by impaired adduction of the left eye with abducting nystagmus of the contralateral eye on lateral gaze. Thus, the patient demonstrated impairment of both abduction and adduction of the left eye through distinct ocular motor abnormalities. In addition, left-sided ptosis and miosis developed, consistent with left-sided Horner’s syndrome. The NIHSS score increased from 7 on admission to 9 on the first hospital day [4]. Despite the evolution of these focal neurological findings, there was no deterioration in his level of consciousness.

Repeat non-contrast CT of the brain performed the following day demonstrated no significant interval change in the volume of the acute left pontine intraparenchymal hemorrhage, measuring approximately 2.8 cc compared with 3.0 cc on the initial study, with minimal decrease in hematoma density. There was no ventricular dilatation or midline shift.

Transthoracic echocardiography demonstrated normal left ventricular dimensions, with global hypocontractility and a calculated left ventricular ejection fraction of 46%. The right ventricle was normal in dimension with hypocontractility from the mid-ventricle to the apex. Grade III left ventricular diastolic dysfunction was present. Mild mitral, tricuspid, and pulmonic regurgitation were also noted, with a normal estimated pulmonary artery systolic pressure of 22 mmHg and no pericardial effusion.

Given the hyperacute onset of focal neurological deficits and the demonstration of an acute pontine hemorrhage on non-contrast CT, a hemorrhagic cerebrovascular event was established as the underlying etiology. The principal diagnostic consideration was therefore the syndromic and anatomical localization of the lesion based on the combination of ipsilateral cranial nerve abnormalities and contralateral long-tract findings.

Millard-Gubler syndrome was considered because of the combination of ipsilateral peripheral facial palsy and contralateral hemiparesis [5]. However, this syndrome typically results from a ventral pontine lesion and would not adequately account for the additional internuclear ophthalmoplegia and ipsilateral Horner’s syndrome observed in this patient.

Raymond syndrome, characterized by ipsilateral abducens nerve palsy with contralateral hemiparesis, was also considered [6]. However, it does not characteristically involve the facial nerve nucleus or fascicle and therefore would not explain the ipsilateral peripheral facial palsy. The subsequent development of internuclear ophthalmoplegia and Horner’s syndrome further argued against this localization.

Lateral pontine syndromes were considered less likely because of the absence of vestibulocochlear symptoms, cerebellar signs, and facial sensory deficits, together with the presence of ocular motor findings localizing to the pontine tegmentum [2].

The combination of ipsilateral abducens palsy, ipsilateral peripheral facial palsy, internuclear ophthalmoplegia, ipsilateral Horner’s syndrome, and contralateral motor and sensory deficits was therefore most consistent with an extended Foville syndrome arising from the left pontine tegmentum, concordant with the anatomical location of the hemorrhage demonstrated on CT [1,2].

The patient was managed conservatively with close neurological monitoring and blood pressure control using antihypertensive therapy. Hypertonic saline was administered for medical management of cerebral edema. Following the progression of neurological deficits on the first hospital day, repeat non-contrast CT demonstrated no significant interval increase in the volume of the pontine hemorrhage. Neurosurgical intervention was not indicated.

He was also given atorvastatin 40 mg once daily at bedtime and citicoline 1 g twice daily. Further vascular imaging and magnetic resonance imaging were not pursued during the acute admission because the hemorrhagic lesion was clearly demonstrated on CT and subsequent imaging showed no significant hematoma expansion. Following clinical stabilization, the patient was enrolled in a structured rehabilitation program.

The patient remained clinically stable throughout the remainder of his hospital course, without deterioration in level of consciousness or development of additional neurological deficits after the first hospital day. He was subsequently discharged on antihypertensive therapy, atorvastatin, and citicoline, with arrangements for continued outpatient rehabilitation.

At 1.5 months postictus, the patient had persistent focal neurological deficits but demonstrated functional improvement with rehabilitation. He was ambulatory with assistance and had a modified Rankin Scale (mRS) score of 3 [7,8].

At the latest follow-up eight months postictus, further neurological and functional improvement was documented. His NIHSS score had decreased to 6 [4], as compared with 9 on the first hospital day. Residual neurological findings included left lateral rectus paresis, left peripheral facial palsy, moderate dysarthria, mild right hemiparesis with muscle strength of 4+/5, and right hemisensory loss. His mRS score had improved to 2 [7,8], and he was able to ambulate independently without assistance.

## Discussion

Foville syndrome is a classic but infrequently encountered brainstem syndrome resulting from lesions of the pontine tegmentum [1,2]. It is traditionally characterized by ipsilateral abducens nerve palsy, ipsilateral peripheral facial nerve palsy, and contralateral hemiparesis, reflecting involvement of the abducens nucleus or fascicle, facial nerve fibers, and corticospinal tracts, respectively [1,2]. Because of the compact anatomical organization of the pons, small lesions in this region may produce a complex constellation of neurological deficits, making careful clinical localization essential [2].

In the present case, the initial combination of left lateral rectus paresis, left peripheral facial palsy, moderate dysarthria, right hemiparesis, and right hemisensory loss was anatomically consistent with involvement of the left pontine tegmentum. The subsequent progression to complete left abducens palsy, worsening contralateral sensory loss, and development of left internuclear ophthalmoplegia and Horner’s syndrome on the first hospital day further refined the localization. Internuclear ophthalmoplegia indicated involvement of the medial longitudinal fasciculus, while ipsilateral Horner’s syndrome suggested disruption of descending hypothalamospinal sympathetic fibers [2]. The corresponding increase in NIHSS from 7 to 9 provided an objective measure of the early neurological progression [4]. This evolution underscores the importance of serial neurological examinations in patients with brainstem stroke, as additional localizing signs may emerge after the initial presentation.

The ocular motor findings merit further neuroanatomical distinction. On admission, the left eye was esotropic in primary gaze with left lateral rectus paresis, while vertical gaze remained intact. By the first hospital day, the lateral rectus paresis had progressed to complete palsy with loss of abduction, accompanied by newly developed left internuclear ophthalmoplegia causing impaired adduction of the same eye. Although this combination resulted in severe limitation of horizontal ocular motility of the left eye, an ipsilateral conjugate horizontal gaze palsy was not documented. Therefore, the findings are better interpreted as combined involvement of the left abducens pathway and medial longitudinal fasciculus rather than definitively classified as classic one-and-a-half syndrome.

The contralateral hemisensory deficit suggested additional involvement of ascending sensory pathways within the pons, including the medial lemniscus and/or spinothalamic tract, further supporting an extended form of Foville syndrome [1,2]. The overall constellation could not be adequately explained by ventral pontine syndromes, such as Millard-Gubler or Raymond syndrome, particularly after the emergence of internuclear ophthalmoplegia and ipsilateral Horner’s syndrome [5,6]. Clinicoradiologic correlation with the focal left pontine tegmental hemorrhage therefore supported the diagnosis of extended Foville syndrome [1,2].

Although ischemic stroke is a recognized vascular cause of Foville syndrome, hemorrhagic lesions of the pontine tegmentum are less frequently described [1]. Pontine hemorrhages may be associated with severe neurological impairment and mortality, particularly with increasing hematoma size and more extensive involvement [3,9]. CT findings, including hematoma size and anatomical extent, have also been associated with clinical outcome in primary pontine hemorrhage [10]. In contrast, the relatively focal tegmental hemorrhage in this patient produced highly localizing deficits with preservation of consciousness. Non-contrast CT demonstrated a focal hemorrhage anatomically concordant with the neurological findings and showed no significant hematoma expansion on repeat imaging.

The patient’s subsequent course also demonstrates that persistent focal brainstem deficits do not necessarily preclude meaningful functional recovery. At 1.5 months postictus, he remained ambulatory with assistance with an mRS score of 3 [7,8]. By 8 months, his NIHSS had improved to 6 [4] and his mRS to 2 [7,8], with independent ambulation despite residual ocular motor, facial, motor, sensory, and speech deficits. This longitudinal improvement emphasizes the value of continued rehabilitation and longer-term follow-up when assessing functional outcomes after focal pontine hemorrhage.

This case highlights the enduring relevance of bedside neurological examination in the era of advanced neuroimaging. Recognition of evolving ocular motor, cranial nerve, autonomic, motor, and sensory findings allowed increasingly precise anatomical localization, while serial NIHSS and mRS assessments documented the patient’s neurological and functional trajectory [4,7,8]. For trainees and clinicians, extended Foville syndrome provides a valuable illustration of how detailed neuroanatomical examination, serial reassessment, and targeted neuroimaging complement one another in the evaluation of brainstem stroke.

## Conclusions

This case illustrates an extended Foville syndrome secondary to a focal pontine tegmental hemorrhage, characterized by evolving and anatomically localizing cranial nerve, ocular motor, autonomic, motor, and sensory deficits. Serial neurological examination demonstrated progression from left lateral rectus paresis to complete palsy with loss of abduction, followed by development of ipsilateral internuclear ophthalmoplegia and Horner’s syndrome. The ocular motor findings were consistent with combined involvement of the left abducens pathway and medial longitudinal fasciculus rather than a definitively documented classic one-and-a-half syndrome. Clinicoradiologic correlation with the focal pontine tegmental hemorrhage supported the anatomical localization, while longitudinal NIHSS and mRS assessments demonstrated meaningful neurological and functional recovery over 8 months. This case emphasizes the value of precise bedside neuroanatomical examination, serial reassessment, targeted neuroimaging, and longitudinal follow-up in patients with focal brainstem hemorrhage.
